# A first glance into the black box of life satisfaction surrounding childbearing

**DOI:** 10.1007/s12546-021-09267-z

**Published:** 2021-06-13

**Authors:** Arnstein Aassve, Francesca Luppi, Letizia Mencarini

**Affiliations:** 1 DONDENA Centre for Research on Social Dynamics and Department of Social and Political Science, Bocconi University, Via Sarfatti 25, 20136 Milan, Italy; 2 Department of Statistics, Università Cattolica del Sacro Cuore, Largo Gemelli 1, 20123 Milan, Italy; 3 DONDENA Centre for Research on Social Dynamics and Department of Management, Bocconi University, Via Sarfatti 25, 20136 Milan, Italy

**Keywords:** Life satisfaction, Domains of satisfaction, Childbearing, Longitudinal analysis

## Abstract

The vast majority of studies looking into the relationship between childbearing and subjective well-being use overall measures where respondents either report their general level of happiness or their life satisfaction, leaving substantial doubt about the underlying mechanisms. However, life satisfaction and happiness are intuitively multidimensional concepts, simply because there cannot be only one aspect that affects individuals' well-being. In this study, by considering seventeen specific life satisfaction domains, these features come out very clearly. Whereas all the domains considered matter for the overall life satisfaction, only three of them, namely satisfaction with leisure, health and satisfaction with the partnership, change dramatically surrounding childbearing events. Even though we cannot generalise (since these results stem from one particular panel survey, i.e., Household Income and Labour Dynamics in Australia data), it appears that the typical anticipation and post-child decrease of life satisfaction, so often found in existing studies, stems from changes in these three domains.

## Introduction

With value change and modernisation (Inglehart, 1977, 1989), all Western countries experienced a sustained fertility decline (Billari & Kohler, 2004; Morgan, 2003). With this new fertility regime, proponents of the Second Demographic Transition (Lesthaeghe & van de Kaa, 1986) argue that self-realisation has taken priority over family life and children. Studies have consequently focussed on establishing the relationship between childbearing events and self-reported well-being, with the idea that satisfaction may decline with the onset of children (Aassve et al., 2012, 2015; Clark et al., 2008; Frijters et al., 2011; Le Moglie et al., 2015; Margolis & Myrskylä, 2011; Matysiak et al., 2016; Myrskylä & Margolis, 2014; Pollmann-Schult, 2014). The majority of these studies use a general measure of individuals' reported happiness or overall life satisfaction. Intuitively one would expect one's overall life satisfaction to be derived from a range of sources. Satisfaction with leisure time, with the financial situation, or with work-life balance—just to mention a few—all add up to the broader and more general life satisfaction measure. With an aim of understanding patterns of well-being across time and societies, such an overall measure may suit the purpose well. However, in a life course perspective, individuals' priorities and preferences change according to the life stage in which they find themselves. As for childbearing, which is such a pervasive event in couples' lives, where one is forced to shift efforts and attention towards the new-born child, the satisfaction domains may also shift in significant ways. As policy makers are concerned about declining fertility levels, it is a pertinent need to understand which domains matter more for the satisfaction surrounding childbearing events.

The main contribution of this study is to consider a range of domains of satisfaction, all of which are being held up against childbearing events. Existing studies, based on overall measures of individuals' reported life satisfaction, show an upward trend prior to the actual childbearing event (and often this takes place prior to the date of conception), and then, to taper off after some years. These effects, respectively, are typically interpreted as processes of anticipation and adaptation. We use the Household Income and Labour Dynamics in Australia (HILDA), where one unique feature is that it has repeated recordings over time of satisfaction across several satisfaction domains. In line with previous studies (Clark et al., 2008; Le Moglie et al., 2018; Margolis & Myrskylä, 2011; Myrskylä & Margolis, 2014; Pollmann-Schult, 2014), we estimate a series of fixed effect models, where reported satisfaction in these domains are used as dependent variables, while childbearing events are incorporated as explanatory variables—together with a set of control variables. We complement the standard fixed effect regression with a Blinder-Oaxaca (1973) decomposition exercise. This approach provides statistical evidence as to which domains matter more in driving the overall life satisfaction surrounding the childbearing event. When comparing overall satisfaction with those of the domains, we find that only a limited number of them react to the childbearing event. For the majority of the domains, childbearing has little or no impact on the reported satisfaction. The study consequently brings insights into why life-satisfaction changes surrounding childbearing.

## Background

### Subjective well-being and childbearing

There is now a number of longitudinal studies focusing on the dynamics of Subjective Well-being (henceforth, SWB) surrounding childbearing events. In the majority of cases, SWB is measured through reported happiness, or overall life satisfaction. In general, independent of the measure used, it tends to increase prior to the actual childbearing event itself, a feature typically referred to as an anticipation effect. It is then followed by a significant decline, especially during the first year of life of the child—a pattern that is again rather robust across Western countries where panel surveys are available. Clark et al. (2008) and Le Moglie et al. (2018), using the German socio-economic panel (SOEP), Myrskyla and Margolis (2014) using both the SOEP and the British Household Panel Survey (BHPS), Frijters et al. (2011) and Matysiak et al. (2016), using the Australian household panel (HILDA), all show clear positive anticipation with respect to the childbearing event, followed by a decrease of SWB in the period after the event took place.

The relationship between childbearing and SWB depends on several preconditions and mediating factors (Kohler & Mencarini, 2016). For instance, SWB trajectories vary by the parents’ age, gender, and socio-economic status (Le Moglie et al., 2018; Margolis & Myrskylä, 2011; Myrskylä & Margolis, 2014), and, to some extent, the country where the parents live (Aassve et al., 2012, 2015). Studies also show that the SWB trajectory associated with the second child is different from the first (Le Moglie et al., 2015; Myrskyla & Margolis, 2014). As prospective parents would have no experience about the impact of childbearing the first time around, they may exaggerate the potential positive feelings about parenthood, and possibly underestimate the upheavals the addition of a new young family member entails. As Myrskyla and Margolis (2014) point out, positive expectations before the onset of parenthood are frequently overoptimistic. The SWB trajectory surrounding the second child is less pronounced but also more heterogeneous across samples.

Making expectations about one's future with a child is clearly part of the planning process that individuals make. Future parents aim to predict consequences of parenthood along several life spheres (work, couple relationship, leisure time, health, etc.). This means that the way individuals emotionally react to childbearing depends on the match (or perhaps mismatch) between expectations and the eventual reality when childbearing takes place. The anticipation of the potential consequences in terms of labour market involvement, often begins early, especially for women (Bass, 2014). As several studies have shown, the transition to parenthood leads to divergent gender paths both in terms of the pay-gap and career opportunities (Grunow et al., 2012; McDonald, 2000). Moreover, women, more than men, consider their fertility intentions when making career choices, and this is especially so among those from a higher socio-economic background. Where higher education delays childbearing, it also brings about better security in terms of career prospects and higher economic resources. Women’s concern about work-family balance is justified by the fact that they usually become the primary caregiver of their children (Baxter et al., 2015; Cowan & Cowan, 1992; Craig et al., 2010; Goldberg & Perry-Jenkins, 2004). Not surprisingly, difficulties in reconciling work and family after the first childbirth are one of the main causes of the decline of mothers’ SWB, at least in the short run (Matysiak et al., 2016). In addition to these factors, the personality of the child itself plays a role. For instance, a child's sleep patterns will necessarily affect the well-being of the parents. Likewise, health problems will potentially affect parental well-being (Brehaut et al., 2011; Davis et al., 2009).

### Overall life satisfaction and its domains

Research in psychology suggests that SWB encompasses both an emotional dimension—i.e., positive and negative affects—and a cognitive dimension—i.e., life satisfaction (Andrews & Withey, 1974). Life satisfaction is an evaluative judgment on one’s life, related to the immediate or very recent context (Schwarz & Strack, 1991). There is an extensive literature concerning the association between life satisfaction and its domains (for a review see Lance et al., 1989). It derives from the simple fact that life satisfaction is necessarily multidimensional. The intuition is simple. Individuals have various needs, and their overall satisfaction depends on the extent to which those needs are satisfied. In social psychology there is consequently an approach known as the “domains of life” (Cummins, 1996; Saris & Ferligoj, 1995; Veenhoven, 1996). It posits that overall well-being depends on satisfaction with each of several life spheres (Campbell et al., 1976).

The domain specific approach prompts individuals' memories about that particular domain, which they often find easier than expressing a precise value of overall satisfaction (Pavot & Diener, 2008; Bargh, 1989; Schwarz et al. 1987). Whereas individuals derive satisfaction from various sources, it is not obvious which domains weigh more (or less) towards overall SWB. The relative importance depends on life events, values, pursued goals (Kasser & Ryan, 1996; Oishi et al., 1999), expectations (Veenhoven, 1996) and life stages (Cantor & Blanton, 1996). Obviously, there is heterogeneity in how those elements matter for individuals (Oishi et al., 1999; Trauer & Mackinnon, 2001; Wu, 2009).

Certain domains have been found particularly relevant for describing the overall SWB (Cummins, 1996). These are satisfaction with health, family, social relationships, leisure-time, work, sex, income, housing, safety, self-worth, and education (Argyle, 2001; Costa, 2008; Greenley et al., 1997; Headey & Wearing, 1992; Praag et al., 2003). Over the life course, individuals change their perception about which life domains are more important for their overall wellbeing (Cantor & Sanderson, 1999; Diener et al., 1999). For example, satisfaction with the couple relationship weighs more strongly during the early stages of the family formation process (Oishi et al., 1999), while health becomes a more important domain in old age (Steptoe et al., 2015). However, independently from the stage of life the individual is experiencing, two domains relate more strongly with overall measures of SWB: social relationships and work (Argyle & Martin, 1991). Social relationships are themselves sources of material help and social support, and they help prevent individuals from experiencing illness and mental distress. However, social relationships can be also sources of dissatisfaction. For example, the partners’ relationship can be one of the strongest sources of conflict and therefore yield low SWB (Argyle & Furnham, 1983). The work domain is relevant simply because it is such a dominant component of an individual’s identity (Furnham, 1991). Likewise, leisure is a source of intrinsic satisfaction (Veroff et al., 1981), because leisure activities tends to positively affect one’s self esteem (Kabanoff, 1982). Moreover, both work and leisure are important sources of social relationships, thereby enhancing social satisfaction.

The question of how satisfaction with specific life domains may change with the arrival of a child has been partially addressed in some studies. None of them, however, aim to assess the relative importance of each domain in describing the trend of parents’ overall life satisfaction across the transition to parenthood. Krämer and Rodgers (2019) explore German SOEP (Socio Economic Panel study) data, and in particular how satisfaction with the overall life and some life domains (life, family life, health, sleep, job, housework, household income, personal income, leisure and dwelling) change and adapt to the arrival of the first child. They found that satisfaction with personal income significantly declines for mothers after the birth of the child, but not for fathers; satisfaction with sleep also declines for both parents. Bernardi and colleagues (2017) find that in Germany (using SOEP data) mothers more than fathers decrease their satisfaction with job and leisure time after the transition to parenthood. Accordingly, a UK study (Georgellis et al., 2012) finds that the birth of the first child has a long-lasting negative effect on job satisfaction for both mothers and fathers. These results might be explained by the following mechanisms. For women, childbearing brings about an interruption to their work career, and as such, childbearing may affect mothers’ career prospects, while the “second-shift” may increase work-family conflict (Matysiak et al., 2016). Moreover, time for leisure also becomes reduced, as mothers have to adjust to the child's needs, especially during the period when the child is very young. Fathers on the other hand, continue to work in most cases, but they may become more sensitive to the financial domain, as they are concerned with guaranteeing an adequate standard of life to their enlarged family. However, compared to mothers, they seem to be favoured in terms of domestic responsibilities and leisure (e.g., Yavorsky et al., 2015).

Additionally, it has been shown that receiving support from relatives, and especially from the new grandparents, is important during the period surrounding childbirth and the time immediately afterwards. Having children and not having informal help from grandparents, for example, may even worsen mothers’ satisfaction with their work-family balance (Arpino & Luppi, 2020). Thus, satisfaction with family relationships is important, and can potentially change during the period before and after childbirth (Liefbroer, 2005; Melender & Lauri, 2002).

Among family relationships, the couple relationship is certainly the one more affected by the arrival of a child. Building and maintaining a strong couple intimate relationship is an important element in early adult life, but it becomes even more crucial when planning to have a child. Its relevance continues once the child is born as parents have to adjust their commitments to work and family tasks, which may bring about conflict and reduce marital satisfaction (Doss et al., 2009; Keizer, 2013; Twenge et al., 2003). However, the housework domain might matter more for mother’s SWB than for fathers’, because she usually takes most of the domestic responsibility after the childbirth. In fact, evidence in the literature shows that women—but not men—who perceive not having an equal share of domestic workload with the partner, show a lower relationship satisfaction if compared to those reporting an equal share (Mikula et al., 2012, Ruppanner et al., 2017). In general, the couple’s overall lifestyle changes after the arrival of the newborn, and this may trigger stress for both parents (Condon et al., 2004). Finally, childbearing can also imply significant changes to the mother’s physical health (Kline et al., 1998), declining their perceived health status, which in turn may negatively affect women’s psychological well-being (Webb et al., 2008).

## Data, sample and variables

We use thirteen waves of data from 2001 to 2013 of the Household Income and Labour Dynamics in Australia survey (HILDA). It is a representative sample of Australian households collecting information about family and labour dynamics, economic and subjective well-being on all the adult members of the households. The original sample at the first wave (2001) was made of 7682 households (around 20,000 individuals) and topped up in 2011. A unique part of the HILDA is that it includes repeated measures of overall life satisfaction and satisfaction with seventeen specific domains. These are (1) satisfaction with the relationship with the partner, (2) Leisure time, (3) Work-Family balance, (4) Employment prospects, (5) Financial situation, (6) Health, (7) Work, (8) Pay, (9) Job Security, (10) Working hours, (11) The job in general, (12) The home, (13) Safety, (14) Community, (15) Neighbourhood, (16) Relationship with parents, and (17) Relationship with the child. Satisfaction with the domains is asked yearly, through the question “How satisfied are you with [the domain]?” on an 11-point scale ranging from zero (completely unsatisfied) to 10 (completely satisfied).

We consider men and women who experienced the first birth and potentially the second child. The final sample is made up of 1061 women (aged 18–50) and 946 men (aged 18–60) at the year of the birth of their first child, and 904 women (aged 18–50) and 750 men (aged 18–60) at the year of the birth of their second child. Individuals are followed over a period of 9 years, from 4 years before the year of birth of the child to 4 years after. The year prior to the childbirth is taken as the pregnancy year. Among those experiencing the first birth during the survey, about 60% also experience the second birth in the subsequent 4 years. The sample is unbalanced due to attrition resulting from missing values on either the satisfaction variables or key explanatory variables.

We include similar control variables as used in previous studies. Other control variables traditionally included in the literature on fertility are also available. Age is measured by five age classes (less or equal than 25; 26–30; 31–35; 36–40; more than 40), while education is divided in three classes: (1) primary (those who does not reach the end of the secondary school), (2) secondary and advanced diploma, tertiary and (3) postgraduate education. Income refers to equivalent disposable household income (net of taxation and divided by the number of household members). Health status is measured on a five-point scale and refers to objective limitations in daily life activities because of health problems. Work status is derived from working hours per week and we distinguish those working part-time, full-time, and more than 40 h per week, and those not working at all. The percentage of unemployed women or men is small (around 4–5%). Consequently, inactive and unemployed respondents are lumped together. While there is not much variability within the distribution of working hours over time in the sample of men, the birth of the first child strongly increases the percentage of inactive women (from 12% 2 years before the birth to 41% the year after the birth) or women working less than full-time hours (from 17 to 45% in the same period).

Finally, we control for whether other life events happen during the period of study. In particular, we include a control for the occurrence of another pregnancy and another birth (first, second or third order), for the presence of another (first, second or third) child aged 1 year or more, and for the respondent getting married.

## Methods

We undertake fixed effects regressions—separate for women and men for each of the domains of satisfaction. We include time dummies for the 4 years before and the 4 years after the birth of the child, using a, reference category 4 years before the event. The model for the individual’s satisfaction over time (S_it_) is defined as:

1 $${\text{S}}_{it} = {\text{a}}_{i} + {\text{bX}}_{it} + {\text{T}}_{t} +\upvarepsilon _{it}$$

where T_*t*_ refers to the time dummies, X_*it*_ is the vector of the time-dependent covariates, a_*i*_ captures individual level unobserved heterogeneity and ε_*it*_ is the vector of the residuals. From its estimation, we show the time paths of the satisfaction variables to see to what extent the domains are similar (or differ) to the overall life satisfaction path. They consequently provide an indication of the domains' sensitivity with respect to the childbearing events. The models are estimated, and the trajectories plotted, by gender and birth order.

In Sect. 5 we present the results from a Blinder and Oaxaca (1973) decomposition exercise. In particular, the dependent variable is overall life satisfaction, whereas groups are defined over two time points. In the first set of analyses, we compare the year of the pregnancy and 1 year after. In the follow up, we also compare the year of childbirth, and subsequent periods. The explanatory variables of interest are the full set of domain specific satisfaction. We are consequently able to establish the extent to which the gap is explained by the differences in the mean values of the satisfaction domains.

## Results from fixed effect regression

**Fig. 1:**
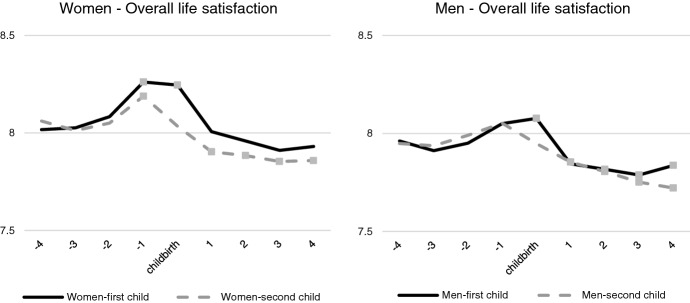
Trajectories over time of *overall life satisfaction*, for women and men, across the transition to the first and the second child (predicted values from multivariate regression with fixed effects. Reference categories: 4 years before childbirth; 31–35 years old; primary education; working full-time). *Note 1*: control variables are age classes, working conditions—i.e., inactive/unemployed; part-time less than 36 h/week; full-time 36–40 h/week; full-time more than 40 h/week, equivalent household income, health conditions, experience of separation/divorce, experience of death of partner/close relative or friend, pregnancy of another child, birth of another child, age classes, level of education. *Note 2*: dots indicate the coefficient is significant at least at *p* = 0.05

**Fig. 2:**
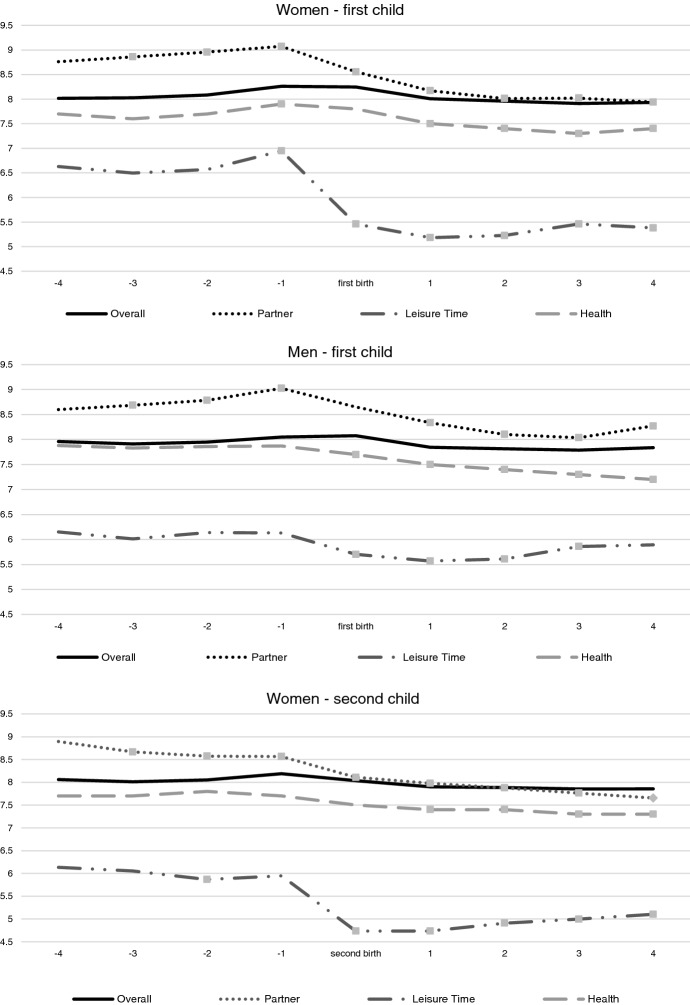

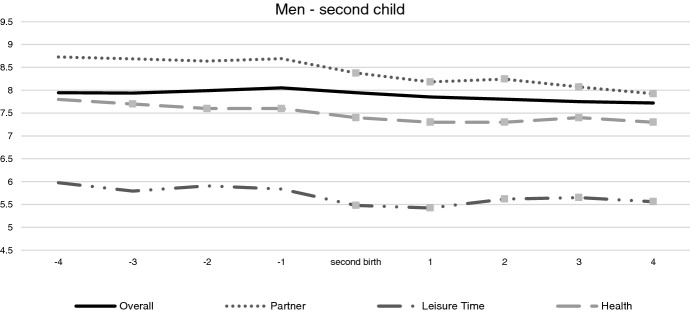
Trajectories over time of three domains of satisfaction (partner relationship, health and leisure time) and of the overall life satisfaction, for women and men, across the transition to the first and second child (fixed effects, controlling for socio-demographic characteristics and other life events. Reference categories: 4 years before childbirth; 31–35 years old; primary education; working full-time). *Note 1*: control variables are age classes, working conditions—i.e., inactive/unemployed; part-time less than 36 h/week; full-time 36–40 h/week; full-time more than 40 h/week, equivalent household income, health conditions, experience of separation/divorce, experience of death of partner/close relative or friend, pregnancy of another child, birth of another child, age classes, level of education. *Note 2*: dots indicate the coefficient is significant at least at *p* = 0.05

From the fixed effect estimation,^1^ we start by plotting the estimated overall life satisfaction trends for women and men around the birth of the first and second child (Figs. 1 and 2). The dots indicate statistical significance at the 5% level, at least. The overall satisfaction shape is consistent with the main results in the literature. For the transition to first child, both women and men show an anticipation effect that is manifested by an increase in estimated life satisfaction. After the birth of the child, satisfaction decreases, with a slight tendency of recovery at the fourth year. Anticipation is more evident for women than for men. For mothers, the increase in life satisfaction is significant for the year before the birth of the child. The year of the first childbirth is associated with a higher level of life satisfaction for both the parents, while it is not the case for the second birth. After the first birth, life satisfaction declines for both genders, but the decrease is significant only for men. After the second birth, the decrease is strong and significant for both women and men.

**Fig. 3:**
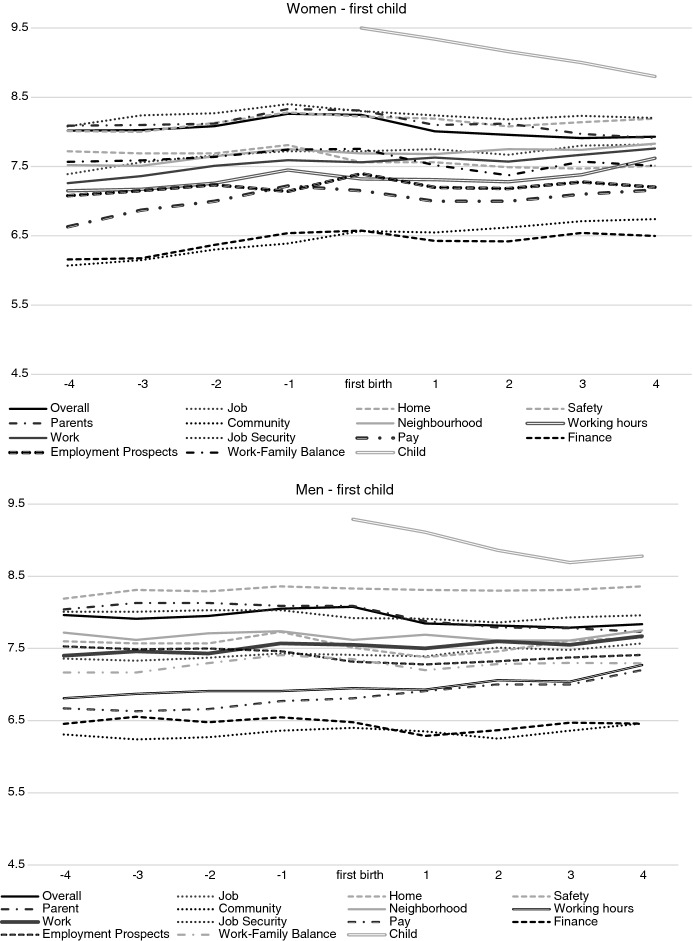
Trajectories over time of other domains of satisfaction and of the overall life satisfaction, for women and men, across the transition to the first child (fixed effects, controlling for socio-demographic characteristics and other life events. Reference categories: 4 years before childbirth; 31–35 years old; primary education; working full-time). Note 1: control variables are age classes, working conditions—i.e., inactive/unemployed; part-time less than 36 h/week; full-time 36–40 h/week; full-time more than 40 h/week, equivalent household income, health conditions, experience of separation/divorce, experience of death of partner/close relative or friend, pregnancy of another child, birth of another child, age classes, level of education

**Fig. 4:**
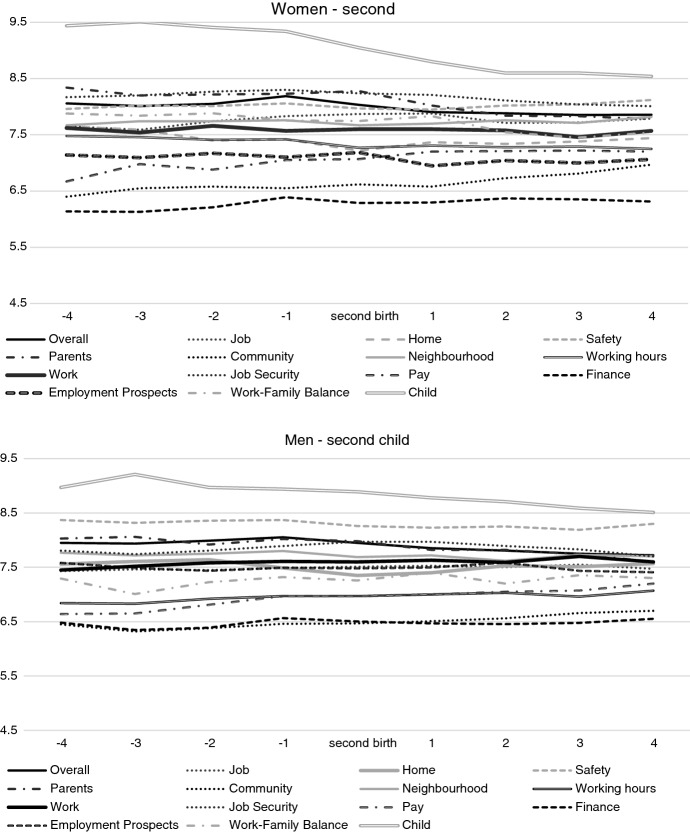
Trajectories over time of other domains of satisfaction and of the overall life satisfaction, for women and men, across the transition to the second child (fixed effects, controlling for socio-demographic characteristics and other life events. (Reference categories: 4 years before childbirth; 31–35 years old; primary education; working full-time). *Note 1*: control variables are age classes, working conditions—i.e., inactive/unemployed; part-time less than 36 h/week; full-time 36–40 h/week; full-time more than 40 h/week, equivalent household income, health conditions, experience of separation/divorce, experience of death of partner/close relative or friend, pregnancy of another child, birth of another child, age classes, level of education. *Note 2*: dots indicate the coefficient is significant at least at *p* = 0.05

In order to compare the trend of overall satisfaction with trends in the life domains, we plot the coefficients of the fixed effects models for each of these (see Figs. 1 and 2; Figs. 3 and 4 in Appendix). The first aspect to notice is that the domain specific satisfaction patterns are in many cases at a quite different level compared to overall satisfaction. For the transition to first child, most of the domains do not show significant variations surrounding the childbearing event. Eight of them do not react explicitly to the childbirth event. Some of these domains remain stable across the entire time span (such as the domains of job security and housing), whereas others show a monotonic increase or decrease if compared to 4 years before the birth of the child—thus they cannot easily be associated with the childbirth itself. These latter domains include satisfaction with job, pay, work, working hours, feeling of belonging to the community and neighbourhood. The other eight domains show significant changes in the level that can be associated with the parenting experience. Among them, we find three domains that stand out because they show the typical path of anticipation and decline surrounding the childbearing event. These are satisfaction with the partner relationship; leisure time and health (see Figs. 1 and 2).^2^ Satisfaction with the partner increases more for men before the birth of the first child, whereas the decline afterwards is less pronounced compared to women. As for satisfaction with leisure time, we see a rather dramatic decline for women. The decline for men is also noticeable but compared with women, it is less pronounced. A similar trajectory can be seen for the satisfaction with health, where a slight increase at the pregnancy year—for women, but not for men—is followed by a continuous decline after the birth, for both parents. Importantly, for neither of these domains, do we see any indication that women's satisfaction returns to the original level observed prior to the childbearing event.

When considering the second child, the patterns are less pronounced, though for women, the decline in satisfaction with leisure still stand out. For this domain we see a very sharp decline during the year of pregnancy. From the time of the birth of the second child, we also see an important trend of recovery in this domain, but it never reaches the original level. The other domains show less dramatic trends, though for women, we do see a steady decline in the satisfaction with the partner and health. There is no peak surrounding the childbearing event however, though the decline appears to become sharper one year before the birth event. The systematic loss of satisfaction after the second birth is especially evident for mothers, who do not show adaptation in partners’ relationship, leisure time nor health domains. In this sense, the second child appears as a rather “detrimental event” in terms of mothers' well-being—and for some domains, the decline appears long lasting.

The implication of these estimates is that when considering the shape of overall life satisfaction surrounding first childbirth for women—typically manifested by a positive anticipation effect, then followed by a decline and a return to the original baseline level—the main drivers come from the satisfaction with leisure time, health and the partner relationship. This is not to say that none of the other domains matter, however. Whereas the satisfaction with the work-family balance, the employment prospects, the financial situation, the relationship with the parents and the feeling of safety do not peak surrounding the childbirth, they do change over the observed time period. For instance, the trend of satisfaction with the work-family balance declines strongly for women from 2 years after the birth of the second child, which is when many mothers go back to work. The positive effect for men after the first birth and the evident negative trend for women after the second birth might be due to the fact that, in Australia, women take care of most of the responsibilities for the household tasks—and especially childcare (Baxter et al., 2015; Craig et al., 2010). The norm in Australia, is that in couples with young children, the male partner is regularly the main income provider, and the woman does not work or works part-time (OECD family database, 2012). As a consequence, women’s career prospects are not necessarily greatly affected by the arrival of the first child—because they may in any case expect a lower involvement in the labour market—while the second child, instead, increases the double burden of working mothers, leading to an unforeseen reduction of career dedication or employment opportunities. On the contrary, the negative trend of the satisfaction for men's employment prospects might be due to increasing family needs and priorities, which take time away time from work. This potentially relates to men’s loss of satisfaction with the financial situation at the year after the birth of the first child. Grandparents seem to provide important support to first time mothers’ well-being, especially during the pregnancy and the first year of life of the child. In fact, the satisfaction with the parents’ relationship increases for women during this time. However, after the birth of the child, the satisfaction with the parents’ relationship declines, especially for men. At the same time, consistent with previous literature, social support received by relatives and friends during the pregnancy and the early years of the first child appears to be responsible for the increase in the feeling of safety for both mothers and fathers (Liefbroer, 2005; Melender & Lauri, 2002).

## Decomposition analysis

Following up on the analysis in Sect. 4, we implement here a decomposition analysis for the sample of those becoming parents for the first time. This approach provides statistical evidence as to which satisfaction domain matters for overall life satisfaction. We use the well-known Blinder–Oaxaca decomposition approach (Blinder, 1973; Oaxaca, 1973), where the idea is to establish the relative importance of a set of factors with respect to any outcome variable (Jann, 2008). The approach is widely used in labour economics, where, for instance, one is interested in drivers behind the pay gap between men and women. As always, the method distinguishes the importance of explanatory variables into the explained part (i.e., by group differences on certain explanatory factors), and, in an unexplained part (residuals). In our case, the two groups are represented by individuals at the pregnancy year and one year after the first childbirth, where the dependent variable is overall life satisfaction and the explanatory factors are the domains of life satisfaction. The analysis is undertaken separately for men and women.

Table 1 shows the “Overall” result of the decomposition analysis and the explained part (Endowments), which is the one assessing the relative importance of each domain and is used to interpret differences of overall life satisfaction before and after the arrival of the first child. The mean level of life satisfaction is higher during the pregnancy year for both women and men, and the difference between the two time points is significant. The endowment coefficients are significant in both cases, meaning that differences in life satisfaction are significantly explained by differences in explanatory factors between the two groups. The results in the “Endowments” part support our previous graphical analysis in Sect. 4, where we plotted the predicted satisfaction levels for separate domains based on fixed effect regressions. For men, we see that satisfaction with the partner, leisure time, and health are the domains that matter for overall life satisfaction surrounding the childbearing event. For women, the same domains matter, though we also find that satisfaction with housing matters significantly. In terms of the magnitude, satisfaction with the partner is by far the most important one, followed by satisfaction with health. Satisfaction with leisure time and housing, though significant, matters less than the other two domains.

**Table 1:** Decomposition analysis of the differential in life satisfaction before and after the arrival of the first child explained by differences in satisfaction with life domains (separated models for women and men)

Overall	Men	Women
*Years from the birth*		
Pregnancy year	8.106	8.284
1 year after	7.917	8.115
Decomposition		
Difference	0.189***	0.169***
Endowments	0.236***	0.263***
Coefficients	− 0.011	− 0.154***
Interaction	− 0.036	0.061
*Endowments*		
Satisfaction with		
Relationship with partner	0.079***	0.123***
Work	0.000	0.001
Work-family balance	− 0.001	0.006
Financial situation	0.019	0.016
Employment prospects	0.020	0.012
Job security	0.001	− 0.001
Parents	0.005	− 0.001
Leisure time	0.034***	0.037*
Health	0.038***	0.064***
Neighbourhood	0.006	0.000
Job	0.003	− 0.015
Pay	0.001	0.000
Working hours	0.000	0.000
Community	0.000	− 0.015
Housing	0.015	0.029**
Safety	0.016	0.007

***For *p* = 0.001; **for *p* = 0.01; *for *p* = 0.05

In Tables 2 and 3 we extend the analysis by considering the decomposition of overall life satisfaction by comparing the year of childbirth and subsequent years up to t + 4, and again we do this separately for men and women. As one can see, for both men and women, overall life satisfaction declines over these time periods, and focusing on the reported differences, we see that the decline is more pronounced for women, though it stabilises after t + 3. When looking to the domains, we find again that the same three stand out: Satisfaction with the partner, leisure time and health—though for women, satisfaction with leisure time does not contribute to explaining the difference between men and women for the first time period. In terms of relative importance (in explaining the change in overall life satisfaction), we see that over time, satisfaction with the partner exceeds that of health and leisure time.

**Table 2:** Decomposition analysis of the differential in life satisfaction between the year of the birth of the first child and the subsequent years, explained by differences in satisfaction with life domains (models for women)

Women	t = 0 versus t = 1		t = 0 versus t = 2		t = 0 versus t = 3		t = 0 versus t = 4
*Overall*							
t0	8.301***	t0	8.301***	t0	8.301***	t0	8.301***
t + 1	8.115***	t + 2	8.063***	t + 3	7.925***	t + 4	7.955***
Difference	0.186***	Difference	0.238***	Difference	0.376***	Difference	0.346***
Endowments	0.102*	Endowments	0.215***	Endowments	0.234***	Endowments	0.246***
Coefficients	0.055	Coefficients	0.028	Coefficients	0.147*	Coefficients	0.101
Interaction	0.029	Interaction	− 0.006	Interaction	− 0.005	Interaction	0.000
*Endowments*							
Satisfaction with							
Relationship with partner	0.038*	Relationship with partner	0.047***	Relationship with partner	0.071***	Relationship with partner	0.099***
Work	0.001	Work	0.001	Work	0.002	Work	0.000
Work-family balance	0.000	Work-family balance	0.000	Work-family balance	0.005	Work-family balance	− 0.004
Financial situation	− 0.001	Financial situation	0.011	Financial situation	0.000	Financial situation	0.013
Employment prospects	0.003	Employment prospects	0.000	Employment prospects	0.008	Employment prospects	0.009
Job security	− 0.001	Job security	0.014	Job security	− 0.003	Job security	− 0.008
Parents	− 0.002	Parents	0.002	Parents	− 0.001	Parents	0.006
Leisure time	0.005	Leisure time	0.041***	Leisure time	0.044**	Leisure time	0.051***
Health	0.056***	Health	0.043***	Health	0.057***	Health	0.047***
Neighbourhood	0.000	Neighbourhood	− 0.002	Neighbourhood	0.005	Neighbourhood	0.004
Job	− 0.007	Job	0.006	Job	0.002	Job	0.008
Pay	0.001	Pay	0.000	Pay	− 0.001	Pay	− 0.004
Working hours	0.000	Working hours	0.001	Working hours	− 0.007	Working hours	0.000
Community	− 0.005	Community	0.000	Community	0.000	Community	0.000
Housing	0.005	Housing	0.022	Housing	0.015	Housing	0.001
Safety	0.008	Safety	0.027	Safety	0.036*	Safety	0.024

***For *p* = 0.001; **for *p* = 0.01; *for *p* = 0.05

**Table 3:** Decomposition analysis of the differential in life satisfaction between the year of the birth of the first child and the subsequent years, explained by differences in satisfaction with life domains (models for men)

Men	t = 0 versus t = 1		t = 0 versus t = 3		t = 0 versus t = 2		t = 0 versus t = 4
*Overall*							
t0	8.159***	t0	8.159***	t0	8.159***	t0	8.159***
t + 1	7.917***	t + 2	7.921***	t + 3	7.895***	t + 4	7.926***
Difference	0.242***	Difference	0.238***	Difference	0.264***	Difference	0.233***
Endowments	0.112**	Endowments	0.159***	Endowments	0.198***	Endowments	0.143***
Coefficients	0.150***	Coefficients	0.117**	Coefficients	0.097	Coefficients	0.129**
Interaction	− 0.020	Interaction	− 0.037	Interaction	− 0.031	Interaction	− 0.039
*Endowments*							
Satisfaction with							
Relationship with partner	0.035***	Relationship with partner	0.057***	Relationship with partner	0.102***	Relationship with partner	0.085***
Work	0.000	Work	0.001	Work	0.000	Work	− 0.004
Work-family balance	− 0.002	Work-family balance	0.001	Work-family balance	− 0.002	Work-family balance	− 0.008
Financial situation	0.010	Financial situation	0.007	Financial situation	0.001	Financial situation	0.001
Employment prospects	0.009	Employment prospects	0.000	Employment prospects	− 0.002	Employment prospects	− 0.001
Job security	0.000	Job security	0.000	Job security	0.003	Job security	0.003
Parents	0.009	Parents	0.001	Parents	− 0.010	Parents	− 0.023*
Leisure time	0.015	Leisure time	0.025*	Leisure time	0.014	Leisure time	0.020
Health	0.020*	Health	0.048***	Health	0.077***	Health	0.082***
Neighbourhood	− 0.006	Neighbourhood	0.000	Neighbourhood	− 0.003	Neighbourhood	− 0.006
Job	0.003	Job	− 0.008	Job	0.005	Job	− 0.011
Pay	0.001	Pay	0.003	Pay	− 0.002	Pay	− 0.001
Working hours	0.002	Working hours	0.000	Working hours	0.000	Working hours	− 0.003
Community	0.000	Community	− 0.001	Community	0.000	Community	0.000
Housing	0.008	Housing	0.016	Housing	0.003	Housing	0.000
Safety	0.008	Safety	0.010	Safety	0.012	Safety	0.009

***For *p* = 0.001; **for *p* = 0.01; *for *p* = 0.05

For men (Table 3), we also see a sharp decline, but there is not much difference across the time periods following the time of childbirth. In other words, the difference between the time of childbirth and other time periods are similar. As for the domains, the most striking difference compared with women is that satisfaction with leisure time is not significant in explaining the decline in men’s overall satisfaction. Satisfaction with the partner, in contrast, becomes stronger across the time period in explaining the decline in overall satisfaction. As for women, men’s reported satisfaction with health is significant in explaining the decline in overall satisfaction.

## Discussion

The vast majority of studies looking into the relationship between childbearing and SWB use overall measures where respondents either report their general level of happiness or their life satisfaction. This literature shows that SWB tends to increase before childbirth and to decrease in the short term after the event. While this literature has caused considerable interest among social scientists, there has been substantial doubt about the underlying mechanisms and the actual meaning once childbearing is reported as affecting individuals' overall well-being, and certainly, there is considerable disagreement about the extent to which children bring about greater happiness and life satisfaction, or not. However, life satisfaction—or happiness—are intuitively multidimensional concepts, simply because there cannot be only one aspect that affects individuals' well-being. In this analysis, by considering specific domains, these features come out very clearly. Whereas all the domains matter for the overall life satisfaction, only three of them, namely satisfaction with leisure, health, and satisfaction with the partnership, change significantly surrounding childbearing events. Even though we cannot generalise (since these results stem from one particular panel survey), it appears that the typical anticipation and decrease of overall satisfaction so often found in existing studies, stems from changes in these three domains. At the same time, all the other domains—and also the individual’s values, aspirations and personality dispositions—are responsible for buffering and smoothing the ups and downs and make the trend of the overall satisfaction flatter.

Another important insight from this analysis is that for women the leisure and partnership relationship domains appear to suffer a relatively long-lasting decline in satisfaction after childbearing, ending lower than the original level observed four years prior to the childbearing event. With decomposition analysis, these conjectures are confirmed. Indeed, the three domains of leisure, health and satisfaction with the partner explain the change in overall life-satisfaction surrounding childbirth. This is an interesting finding, because satisfaction on these two domains does not show adaptation tendencies.

The fact that satisfaction with the partner and leisure are sensitive to childbearing events makes intuitive sense. Childbearing is a joint decision and is experienced by the two partners in the couple. As such, a childbearing event will necessarily involve the partner in important ways. The fact that this domain declines so strongly, suggests firstly, that the presence of children tends to compromise the harmony of the couple, potentially bringing about more conflict especially regarding the division of housework and childcare tasks (Doss et al., 2009; Gallie & Russel, 2008; Keizer, 2013; LaRossa & LaRossa, 1981). Secondly, children are time consuming and impose a tremendous change to the daily chores of the household, naturally reducing leisure time and time for a couple’s intimacy (LaRossa & LaRossa, 1981). The fact that women suffer much more than men in terms of their satisfaction with leisure time suggests that the burden tends to fall on women. However, this idea relates to well-known arguments. Mothers are more exposed to demands from parenting, because they are in charge of the primary childcare (Ross & Van Willigen, 1996; Simon, 1992). Being the primary caregiver implies more challenges in reconciling family and work commitment and, as a consequence, higher indirect costs of childbearing for mothers doing the “second-shift” (Craig & Siminski, 2010; Hochschild and Machung, 1989). Some authors have theorised and empirically tested that high indirect costs of childrearing for mothers might be a cause for lower fertility both at the macro (McDonald, 2001, 2013) and micro level (Campione, 2008; Kalmuss et al., 1992; Ruble et al., 1988). The finding that partner relationship and leisure domains stand out has useful theoretical implications. So far, existing studies based on overall life satisfaction or happiness are rather non-theoretical in the sense that it is difficult to infer the underlying mechanisms for exactly why childbearing events should bring about a change in a general subjective well-being measure.

## Appendix

See Figs. 3, 4 and Tables 4, 5, 6, 7 and 8.

**Table 4:** Number of observations without missing values in the overall life satisfaction and control variables (entire sample), for partnered respondents, employed respondents, and with no missing values on all the six domains of satisfaction (partner relationship, finance, leisure time, employment opportunities, work-family balance, health), by gender and year from the birth of the first and second child

Years from the birth	First child				Second child			
	Entire sample	Partnered	Employed	With all domains of satisfaction	Entire sample	Partnered	Employed	With all domains of satisfaction
*All observations*								
− 4	925	686	808	612	926	789	802	694
− 3	1146	913	1032	848	1083	952	888	790
− 2	1427	1221	1290	1117	1311	1167	975	866
− 1	1730	1587	1472	1368	1520	1395	1140	1058
Birth	2007	1913	1292	1254	1654	1593	1032	1004
1	1729	1557	1290	1174	1392	1238	1009	908
2	1487	1278	1077	938	1181	1039	870	776
3	1258	1075	928	809	1008	868	765	663
4	1080	894	807	676	868	725	666	564
Total	12,789	11,124	9996	8796	10,943	9766	8147	7323
*Men*								
− 4	449	325	402	293	440	370	421	355
− 3	547	427	503	399	505	447	469	414
− 2	691	593	648	554	599	537	563	503
− 1	826	759	759	700	695	649	656	612
Birth	946	930	857	836	750	739	692	677
1	814	742	755	690	637	581	592	538
2	700	614	646	570	538	477	505	451
3	600	525	554	488	446	394	423	373
4	507	428	467	400	388	327	361	304
Total	6080	5343	5591	4930	4998	4521	4682	4227
*Women*								
− 4	476	361	406	319	486	419	381	339
− 3	599	486	529	449	578	505	419	376
− 2	736	628	642	563	712	630	412	363
− 1	904	828	713	668	825	746	484	446
Birth	1061	983	435	418	904	854	340	327
1	915	815	535	484	755	657	417	370
2	787	664	431	368	643	562	365	325
3	658	550	374	321	562	474	342	290
4	573	466	340	276	480	398	305	260
Total	6709	5781	4405	3866	5945	5245	3465	3096

No missing information on the satisfaction with the relationship with the partner

No missing information on the satisfaction with the employment prospects and the work-family balance (surveyed among employed people only)

**Table 5:** Sample distribution around the main socio-demographic characteristics at the year of the birth of the first or second child, by gender

	First child				Second child			
	All observations		With no missing domains satisfaction		All observations		With no missing domains satisfaction	
	Men	Women	Men	Women	Men	Women	Men	Women
Age class								
≤ 20	173	502	69	136	32	157	17	32
21–25	1107	1615	778	774	509	901	374	307
26–30	1719	1946	1435	1267	1085	1582	902	827
31–35	1676	1696	1466	1145	1578	1928	1400	1142
36–40	896	757	773	442	1137	1125	988	634
41–45	505	191	409	102	658	254	546	154
Employment status								
Inactive/unemployed	494	2315	0	0	323	2494	0	0
Part-time	452	1957	366	1666	305	2101	275	1875
Full-time	2135	1583	1874	1424	1727	884	1564	795
More than 40 h/week	2999	854	2686	768	2644	468	2385	417
Level of education								
Primary	912	1146	533	370	669	983	464	265
Secondary	3331	2945	2764	1646	2725	2670	2316	1336
Tertiary	1838	2617	1633	1850	1605	2294	1447	1495
Total	6081	6708	4930	3866	4999	5947	4227	3096

**Table 6:** Sample distribution around the main socio-demographic characteristics at the year of the birth of the first child, by gender and childbirth order

	All observations				With no missing domains satisfaction				All observations				With no missing domains satisfaction			
	Men		Women		Men		Women		Men		Women		Men		Women	
	%	Freq	%	Freq	%	Freq	%	Freq	%	Freq	%	Freq	%	Freq	%	Freq
Age class																
≤ 20	1.6	15	7.9	84	1.1	9	2.4	10	0.4	3	2	18	0.1	1	0	0
21–25	20.2	191	24.6	261	19.1	160	18.7	78	8.3	62	1.5	143	6.5	44	7.3	24
26–30	28.3	286	31.2	331	28.8	241	34.5	144	21.9	164	27.2	246	21.9	148	26.9	88
31–35	29.7	281	24.5	260	31.2	261	31.6	132	37.2	279	35	316	38.4	260	41.9	137
36–40	12.2	125	10	106	13.8	115	11.5	48	20.5	154	17.1	155	21.3	144	20.2	66
41–45	7	66	2.8	19	5.9	50	1.4	6	11.7	88	2.9	26	11.8	80	3.7	12
Employment status																
Inactive/unemployed	9.5	90	59.3	629	0.1	1	0.7	3	7.9	59	63	570	0.1	1	1.5	5
Part-time	7.4	70	22.7	241	8.3	69	55.7	233	5.5	41	27.9	252	6.1	41	73.7	241
Full-time	37.8	358	13.2	140	41.4	346	32.1	134	34	255	6.3	57	36.5	247	17.4	57
More than 40 h/week	45.2	428	4.8	51	50.2	420	11.5	48	52.6	395	2.8	25	57.3	388	7.3	24
Level of education																
Primary	15.3	145	17.2	182	12.1	101	8.1	34	14.1	106	17.3	156	12.9	87	8.9	29
Secondary	54	511	44	466	55.6	465	41.4	173	53.9	404	44.9	406	53.3	361	42.2	138
Tertiary	30.7	290	38.9	412	32.3	270	50.5	211	32	240	37.8	342	33.8	229	48.9	160
Total	100	946	100	1061	100	836	100	418	100	750	100	904	100	677	100	327

**Table 7:** Multivariate regressions with fixed effects for the overall life satisfaction, the relationship with the partner and the leisure time, across the transition to the first child, for women and men (Reference categories: 4 years before childbirth; 31–35 years old; primary education; working full-time)

	Men			Women		
	Overall life satisfaction	Relationship with partner	Leisure time	Overall life satisfaction	Relationship with partner	Leisure time
Years from the birth						
3 years before	− 0.057	0.078	− 0.147	0.013	0.103	− 0.12
2 years before	− 0.024*	0.171**	− 0.025	0.074	0.201**	− 0.039
Pregnancy year	0.067**	0.402***	− 0.039	0.256***	0.322***	0.354***
First birth	0.087	0.023	− 0.471***	0.248***	− 0.187*	− 0.111***
1 year after	− 0.151**	− 0.304***	− 0.602***	0.016	− 0.566***	− 0.137***
2 years after	− 0.184***	− 0.553***	− 0.552***	− 0.027	− 0.72***	− 0.131***
3 years after	− 0.217***	− 0.63***	− 0.296*	− 0.072	− 0.717***	− 0.106***
4 years after	− 0.173*	− 0.406***	− 0.253	− 0.048	− 0.804***	− 0.114***
Age class						
< 25	0.006	− 0.003	− 0.12	0.148*	− 0.009	0.303
26–30	− 0.018	0.049	0.045	0.043	0.071	0.260**
36–40	0.044	− 0.024	− 0.200	− 0.078	− 0.084	− 0.336***
> 40	0.087	− 0.068*	− 0.107	− 0.137	− 0.259	− 0.981***
Highest level of education						
Secondary	− 0.081	0.226	− 0.455	0.048	0.065	0.127
Tertiary	− 0.209	0.314	− 0.350	0.193	0.068	0.461
Employment status						
Inactive/unemployed	− 0.155***	− 0.074***	0.932***	0.071	0.192***	0.905***
Working part-time	− 0.002	− 0.185***	0.465***	− 0.059	0.065	0.728***
Working more than 40 h/week	− 0.031	− 0.136***	− 0.750***	− 0.167***	0.015	− 0.583***
Self-assessed health problems	− 0.238***	− 0.304***	− 0.862	− 0.254	− 0.188***	− 0.596***
Equivalent household income	− 0.000	− 0.000	0.009**	− 0.000	− 0.006	0.013
Other life events						
Second pregnancy	0.148***	0.290	0.279***	0.149***	0.809***	0.412***
Second birth	− 0.025	0.066	− 0.144	0.101	− 0.033	− 0.493
Second child 1 year or more	0.060	0.103	− 0.342***	− 0.126**	− 0.490	− 0.983***
Marriage	0.113***	0.140	0.011	0.054	0.074	0.160
Separation	− 0.210***	− 0.104	0.015	− 0.577***	− 0.121***	− 0.182
Constant	8.439***	8.984***	6.880***	8.288***	8.943***	6.105***
N	945	942	945	1060	1040	1060

Reference categories: 4 years before the birth of the child, age class 31–35, secondary level of education, working full-time

^***^For *p* = 0.001; **for *p* = 0.01: *for *p* = 0.05

**Table 8:** Multivariate regressions with fixed effects for the overall life satisfaction, the relationship with the partner and the leisure time, across the transition to the second child, for women and men (Reference categories: 4 years before childbirth; 31–35 years old; primary education; working full-time)

	Men			Women		
	Overall life satisfaction	Relationship with partner	Leisure time	Overall life satisfaction	Relationship with partner	Leisure time
Years from the birth						
3 years before	− 0.015	− 0.034	− 0.177	− 0.054	− 0.213***	− 0.094
2 years before	0.030	− 0.080	− 0.060	− 0.023	− 0.285***	− 0.115
Pregnancy year	0.089	− 0.017	− 0.124	0.103	− 0.281***	− 0.105
Second birth	− 0.016	− 0.104	− 0.128*	− 0.165	− 0.715***	− 0.235**
1 year after	− 0.114	− 0.324*	− 0.152*	− 0.320**	− 0.871***	− 0.186*
2 years after	− 0.166**	− 0.278	0.078	− 0.353***	− 0.972***	− 0.144*
3 years after	− 0.224***	− 0.483**	0.135	− 0.39***5	− 0.154***	− 0.137
4 years after	− 0.255***	− 0.639***	0.054	− 0.398***	− 0.164***	− 0.133
Age class						
< 25	0.060	− 0.131	− 0.139	0.135	0.074	0.163
26–30	− 0.002	− 0.027	0.018	− 0.025	0.021	0.071
36–40	0.156***	0.067	− 0.029	− 0.04	0.001	− 0.108
> 40	0.164	− 0.066	0.028	− 0.011	0.040	− 0.413
Highest level of education						
Secondary	0.174	− 0.608**	0.497	− 0.233	− 0.232	0.611*
Tertiary	0.181	− 0.826**	0.128	− 0.095	− 0.941*	0.747
Employment condition						
Inactive/unemployed	− 0.071	0.210	0.118	0.052	0.099	0.789***
Working part-time	0.028	− 0.086	0.110	0.023	− 0.017	0.683***
Working more than 40 h/week	− 0.103***	− 0.658	− 2.560	− 0.493	− 0.416	− 1.964***
Self-assessed health problems	− 0.223***	− 0.310***	− 0.149	− 0.248	− 0.240***	− 0.187***
Equivalent household income	0.005	− 0.018	0.006	0.003	0.003	0.017
Other life events						
First pregnancy	0.115*	0.380***	0.280***	0.257***	0.379***	1.139***
First birth	0.188***	0.098	− 0.062	0.236***	0.084	− 0.026
First child 1 year or more	0.207	− 0.245	− 0.368	0.114	− 0.029	− 1.310***
Third pregnancy	0.132	0.224*	− 0.242	0.179**	0.381***	0.250
Third birth	− 0.132***	− 0.160	− 0.024	− 0.025	0.270***	0.072
Third child 1 year or more	0.215***	0.331***	− 0.350**	0.115	− 0.030	− 1.211***
Marriage	− 0.029	0.100	0.164***	0.099***	0.287***	0.372***
Separation	− 0.298***	− 0.224***	− 0.033	− 0.484***	− 0.165***	0.201
Constant	8.071***	9.912***	5.713***	8.551***	9.638***	5.256***
N	750	749	750	904	888	904

Reference categories: 4 years before the birth of the child, age class 31–35, secondary level of education, working full-time

^***^For *p* = 0.001; **for *p* = 0.01: *for *p* = 0.05

## Footnotes

1 Coefficients are available in the Appendix, Tables 7 and 8.

2 The same graph has been plotted for the sub-sample of cases which do not show missing values in any of the domains of life satisfaction (see Appendix, Fig. 4).
